# Cadmium-Induced Kidney Injury: Oxidative Damage as a Unifying Mechanism

**DOI:** 10.3390/biom11111575

**Published:** 2021-10-23

**Authors:** Liang-Jun Yan, Daniel C. Allen

**Affiliations:** Department of Pharmaceutical Sciences, College of Pharmacy, University of North Texas Health Science Center, Fort Worth, TX 76107, USA; danielallen@my.unthsc.edu

**Keywords:** cadmium, kidney injury, renal toxicity, mitochondria, oxidative damage, proximal tubule

## Abstract

Cadmium is a nonessential metal that has heavily polluted the environment due to human activities. It can be absorbed into the human body via the gastrointestinal tract, respiratory tract, and the skin, and can cause chronic damage to the kidneys. The main site where cadmium accumulates and causes damage within the nephrons is the proximal tubule. This accumulation can induce dysfunction of the mitochondrial electron transport chain, leading to electron leakage and production of reactive oxygen species (ROS). Cadmium may also impair the function of NADPH oxidase, resulting in another source of ROS. These ROS together can cause oxidative damage to DNA, proteins, and lipids, triggering epithelial cell death and a decline in kidney function. In this article, we also reviewed evidence that the antioxidant power of plant extracts, herbal medicines, and pharmacological agents could ameliorate cadmium-induced kidney injury. Finally, a model of cadmium-induced kidney injury, centering on the notion that oxidative damage is a unifying mechanism of cadmium renal toxicity, is also presented. Given that cadmium exposure is inevitable, further studies using animal models are warranted for a detailed understanding of the mechanism underlying cadmium induced ROS production, and for the identification of more therapeutic targets.

## 1. Introduction

The kidney is a vital organ that performs critical physiological functions by actively filtering excess fluid and secreting waste products including urea, uric acid, and creatinine [1,2]. It is through the process of filtration and reabsorption that the kidneys maintain homeostasis of water, acid-base and, electrolytes [3]. Moreover, the kidney also secretes hormones that participate in the control and regulation of hemodynamics, red blood cell production, and vitamin D maturation [3]. Under abnormal conditions such as fasting and insulin resistance, the kidney can also make glucose via the gluconeogenic pathway [4,5,6] using noncarbohydrate precursors such as pyruvate, alanine, lactate, and glycerol [7].

The kidney is also vulnerable to injuries caused by numerous challenges such as ischemia [8,9,10,11,12], drug toxicity [13,14,15,16,17,18,19], environmental heavy metal exposure [20,21,22,23,24,25,26,27], hypertension [28,29,30], immune injury [31,32], and diabetes [33,34,35,36]. In terms of environmental risk factors, human kidney disease caused by environmental pollutants and occupational-linked toxins is a major public health issue [37]. Cadmium is a toxic heavy metal mainly derived from chemical stabilizers, pigments, nickel-cadmium batteries, and metal coatings and alloys [38]. It is also a toxic element in cigarettes [38]. Accordingly, contaminated soil, air, drinking water, food chains [39,40], and cigarettes, as well as children’s plastic toys [41], are the major sources of human cadmium exposure. Numerous studies focusing on cadmium toxicity have established that the kidney is a primary organ site for cadmium accumulation [42,43]. Indeed, cadmium exposure has been tightly associated with renal dysfunction and kidney damage, causing polyuria and proteinuria [23,24]. The proximal tubule is the major site of cadmium deposition, accumulation, and damage because of the development of proximal tubular epithelial cell hypertrophy with occurrence of polyuria and proteinuria [44,45,46]. Therefore, it is important to counteract cadmium-induced kidney injury to safeguard kidney function.

## 2. Cadmium Absorption, Transportation, and Accumulation in the Kidney

**Figure 1:**
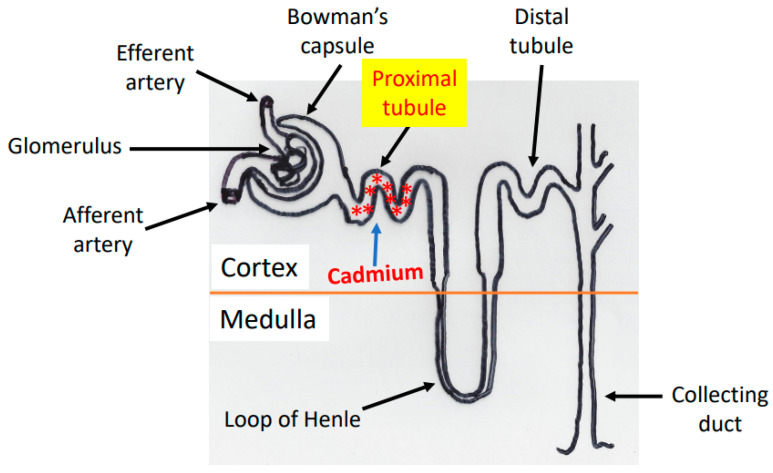
Diagram showing the proximal convoluted tubule as the major site of cadmium accumulation and toxicity in the nephrons “*”.

Cadmium has a high affinity toward thiol groups and can selectively form complexes with proteins and peptides whose cysteine residues are available for cadmium binding [47,48]. After ingestion of cadmium-contaminated water, food, and/or cigarette smoking, cadmium can be absorbed into circulation via the gastrointestinal tract, respiratory tract, or the skin [49,50,51]. Once in the blood, cadmium binds to albumin and other cysteine-containing proteins and peptides such as glutathione [37] and gets transported via many avenues to the liver [37] whereby the heavy metal is then released and induces the expression of metallothionein that then binds tightly to cadmium [52,53]. This binding serves the purpose of detoxification as the cadmium-metallothionein complex is usually considered nontoxic [54]. The cadmium-metallothionein complex can be released into the bloodstream and is then filtered at the glomerulus and reabsorbed by the proximal tubular epithelial cells [55]. This is followed by release of cadmium from the degradation of the cadmium-metallothionein complex [55]. The free form of cadmium in the proximal tubular region of the nephron can then bind to pre-existing renal metallothionein and induce further renal expression of metallothionein [50]. When renal metallothionein is exhausted [56,57], the nonmetallothionein bound cadmium accumulates and induces nephrotoxicity [49,50,51,58,59], primarily in the proximal tubular region (Figure 1) via generation of oxygen free radicals [60,61,62]. As up to 50% of the body’s cadmium pool can deposit in the kidney [37] and the half-life of cadmium in the kidney is approximately 45 years [63,64,65,66,67], cadmium-caused renal toxicity can pose a major threat to human health, particularly in countries where environmental control and regulation are lacking. It should be noted that while the binding of cadmium to metallothionein is a well-established mechanism, other thiol-containing proteins and peptides such as albumin and glutathione can also bind cadmium, leading to functional impairment of these cadmium bound target proteins and peptides [50].

## 3. Cadmium-Induced Animal Models of Kidney Injury

Given the fact that human cadmium exposure is a chronic process at a very low level, any investigation of cadmium renal toxicity would require many years of monitoring and follow-up studies. Therefore, animal models using mice or rats have been widely used to replicate the pathophysiological mechanisms of cadmium renal toxicity [39,40,42,68]. In numerous cases, high doses of cadmium were applied in these animal models to shorten the duration of the studies and facilitate the process of obtaining insights into the mechanisms of cadmium renal toxicity. As mentioned above, studies using rodent models as well as results from human subjects have established that the primary target of cadmium in the nephron is the proximal tubule, whereby cadmium causes overall dysfunction of the epithelial cells [51,69,70], resulting in polyuria and proteinuria [50,51]. There is also an increase in urinary excretion of amino acids, glucose, and electrolytes such as Na^+^. K^+^, and Ca^2+^ [50,51]. Increasing evidence also indicates that a variety of risk factors such as aging [71], malnutrition [72], obesity [73,74,75], and diabetes [27,76] can further superimpose on cadmium renal toxicity and aggravate cadmium-induced renal dysfunction.

It should be stressed that in animal model studies of cadmium renal injury, a variety of doses, routes, and duration of exposures have been performed. The purpose of all these approaches is to try to replicate or recapitulate the toxico-kinetics and underlying mechanisms of long-term, low-level exposure that commonly occur in humans [50].

## 4. Mechanisms of Cadmium-Induced Renal Toxicity

What is the proposed mechanism of cadmium-induced kidney injury? Based on numerous studies, all injurious pathways converge on ROS production and culminate in oxidative stress [77,78,79,80,81], which suggests that oxidative damage is a unifying mechanism of cadmium-induced renal toxicity and injury. We also think that the major sources of ROS causing oxidative damage in this context are mitochondria and NADPH oxidase, described as follows.

## 5. Sources of Reactive Oxygen Species

### 5.1. Mitochondria

**Figure 2:**
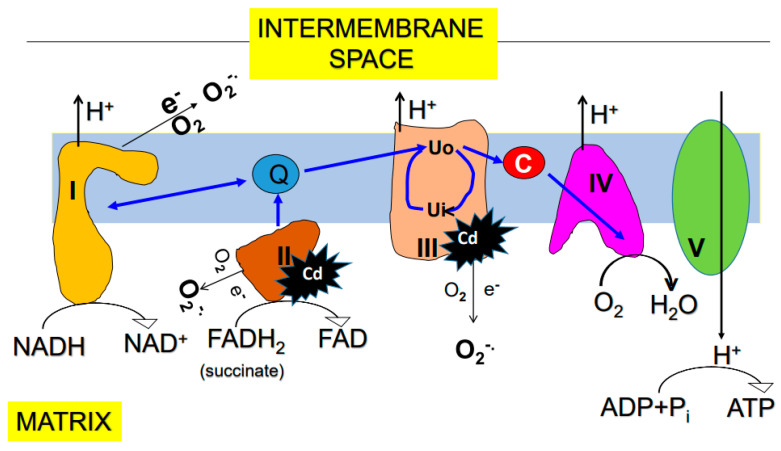
Diagram showing mitochondrial electron transport chain and oxidative phosphorylation. Complexes I, II, and III all can generate superoxide anion. This process can be enhanced by pathophysiological conditions such as cadmium exposure and accumulation. Note that it has been suggested that complexes II and III are the likely sites interacting with cadmium [98].

**Figure 3:**
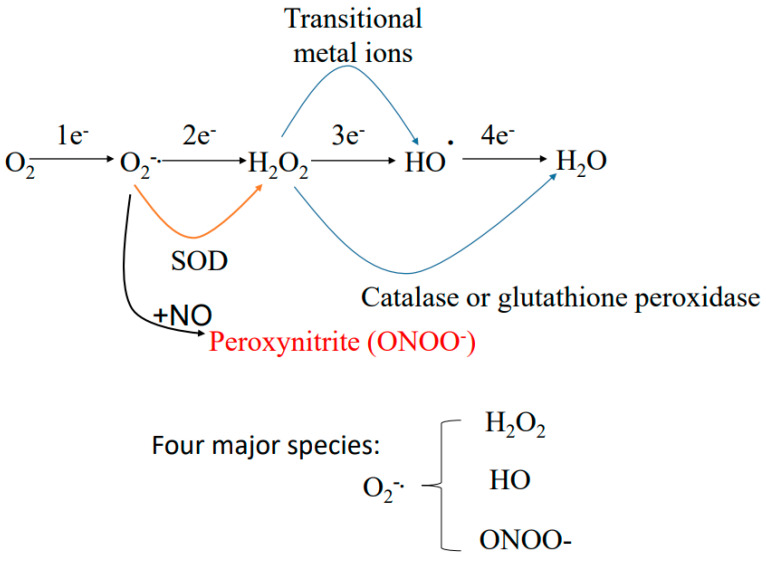
Production of other reactive oxygen species and reactive nitrogen species from the initial species superoxide. Superoxide can be dismutated by superoxide dismutase to form H_2_O_2_, which can be further detoxified by catalase. In the presence of metal ions such as iron, H_2_O_2_ can also generate very reactive species hydroxyl radical. Additionally, superoxide can react with nitric oxide to form peroxynitrite that is also very reactive toward macromolecules.

Mitochondria are well known as the intracellular site of ROS production [82,83,84,85]. Among the electron transport chain components complexes I, II and III have all been established as major sites of ROS production [86,87,88,89]. These sites are not perfect even under normal conditions and can leak electrons out of the transport chain [90,91] (Figure 2). The leaked electrons can then partially reduce oxygen to form superoxide anion, which is the precursor of all other reactive oxygen species including H_2_O_2_, hydroxyl radical, and peroxynitrite [92,93] (Figure 3). Additionally, dihydrolipoamide dehydrogenase involved enzyme complexes such as pyruvate dehydrogenase, α-ketoglutarate dehydrogenase, and branched chain amino acid dehydrogenase can also produce superoxide anion in a variety of experimental and pathological conditions [94,95,96,97].

### 5.2. NADPH Oxidase

**Figure 4:**
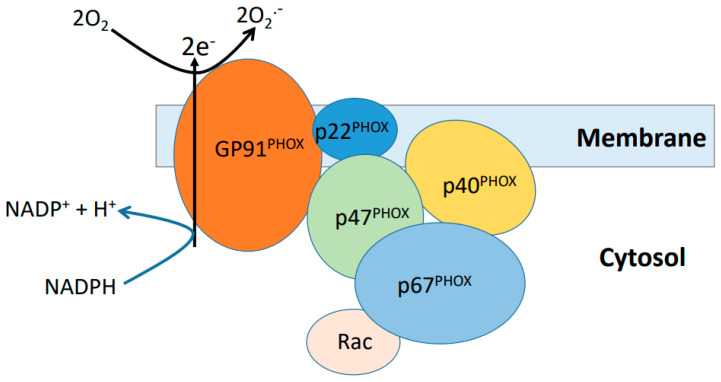
NADPH oxidase assembly and superoxide production at the expense of NADPH. Upon stimulation, each individual subunit of the enzyme is recruited to the membrane and form a membrane-associated complex. Only one subunit GP91 catalyzes partial reduction of oxygen. This figure is adapted from reference [102]. Please not that shown here is NOX2 assembly. For structures and components of other NXO isoforms, please refer reference [103].

NADPH oxidase (NOX) can generate superoxide anion using NADPH as its reducing agent [99]. So far, seven NOXs have been identified (NOX 1-5, Duox1 and DuoX2) [99]. These isoforms differ in many aspects including catalytic oxidase subunit, tissue distribution, intra-cellular location, and mechanisms of regulation [100,101]. All NOXs are composed of multiple subunits. Upon stimulation, these subunits will come together and assemble to form a membrane-associated complex to generate superoxide at the expense of NADPH [102]. Figure 4 shows a representative diagram of NOX assembly upon stimulation whereby the major site of ROS production is the gp91phox subunit with other proteins being the ancillary units required for the regulation and functioning of the whole enzyme complex. It should be noted that Figure 4 only shows the assembly of NOX2. The structural and compositional variations of other NOX isoforms [103] and their potential interaction with cadmium may also play a role in cadmium induced renal toxicity. Under normal conditions, these NOXs function in a beneficial way by regulating kidney metabolism and homeostasis including glucose transport, gluconeogenesis, renal hemodynamics, and electrolyte transport and balance [99]. Under pathophysiological conditions, these NOXs, in particular NOX2 and NOX4 in the kidney, can overgenerate ROS that are damaging to cellular components including DNA, proteins, and lipids, causing cell death and kidney injury [99,104,105,106]. It has been reported that cadmium exposure can increase the expression of NOX1 subunits, leading to increased ROS production from the enzyme [107]. Nevertheless, it is not known exactly which subunit in the NADPH oxidase physically interacts with cadmium at the present time. It should be noted that xanthine oxidase [108,109] and nitric oxide synthase [110,111,112], although not a major source of ROS in the kidney, may also contribute to renal oxidative stress under a variety of pathological and experimental conditions including cadmium exposure. It should also be pointed out that comprehensive evaluations of the roles of NADPH oxidases, xanthine oxidase, and nitric oxide synthase in cadmium-induced kidney injury remain to be conducted.

## 6. Effects of Cadmium on Mitochondrial Function

**Figure 5:**
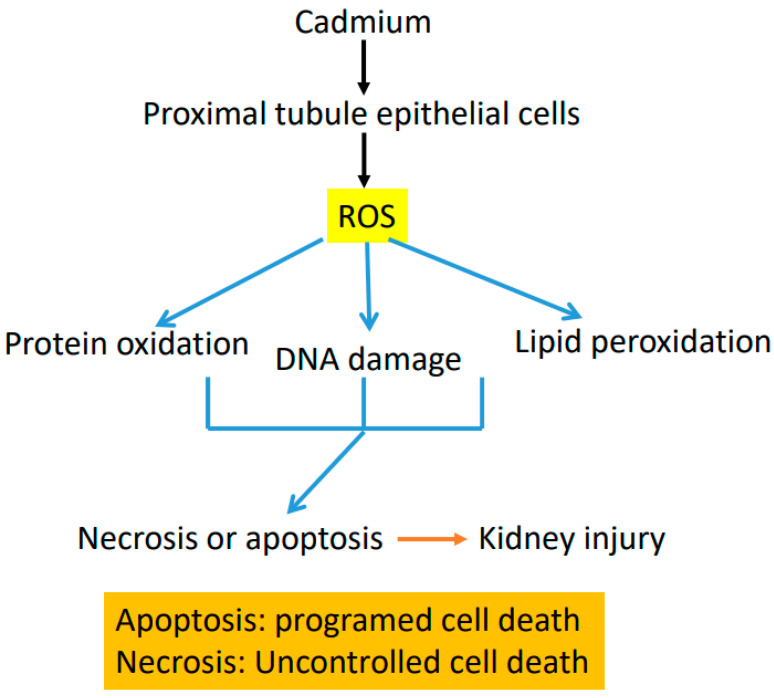
ROS can damage DNA, proteins, and lipids. Damage of the molecules impairs the biological function of each molecule, leading to cell death and kidney injury. Cell death may include both necrosis and apoptosis.

Cadmium can enter mitochondria and accumulate therein [113,114]. This is likely facilitated by mitochondrial membrane channels, and solute molecule carriers and receptors [114]. Once inside the mitochondria, cadmium can bind thiol-containing proteins and impair the corresponding protein function [80]. Studies have demonstrated that upon exposure to cadmium, kidney mitochondria displayed deformation, swelling, and vaculation, concurrent with increased SOD1 expression and decreased SOD2 and catalase expression [115]. Additionally, the anti-apoptotic protein BCL-2 was also found decreased by cadmium exposure [115]; so was the ratio between reduced glutathione and oxidized glutathione [60]. All these could be a generalized cadmium mitochondrial toxicity and the ultimate outcome would be reflected by overproduction of mitochondrial ROS, disruption of mitochondrial metabolic pathways, and impairment of mitochondrial pores, membrane channels and transporters [80]. It has been reported that complex II and complex III may be the major sites impaired by cadmium in the nephrons [98] while the effects of cadmium on proximal tubular mitochondrial complex I (NADH-ubiquinone oxidoreductase) remain unclear. Disruption of all these processes by cadmium would increase mitochondrial ROS production and eventually lead to cell death and kidney injury [80,114,116,117,118,119]. An outline of cadmium induced ROS production, oxidative damage to macromolecules, cell death, and kidney injury is shown in Figure 5, highlighting the concept that oxidative damage is a unifying mechanism of cadmium-induced kidney injury.

## 7. Counteracting Effects of Natural Products, Chemicals and Pharmacological Agents on Cadmium-Induced Kidney Injury

In further support of the notion that oxidative stress and oxidative damage are the universal mechanisms underlying renal toxicity by cadmium, we herein tabulate evidence that numerous natural products such as plant extracts and herbal medicines have been used to counteract the oxidative, deleterious effects of cadmium on the kidney. Many of these studies used cadmium-induced animal models of kidney injury as a platform [120]. Table 1 selectively shows some of the reported plant extracts and herbs as well as exogenous chemicals and pharmacological agents that can attenuate cadmium-induced oxidative stress involved in kidney injury. Additionally, many of these approaches can also induce the activation of endogenous cellular defense systems such as Nrf2, superoxide dismutase, glutathione peroxidase, and catalase [74,121,122,123,124]. Some studies using kidney cell lines such as HEK293 are also included in the table. It should be pointed out that among all the compounds and chemicals listed in Table 1, it is very difficult to identify which one would be the most efficient in terms of combating confirmed cadmium-induced kidney injury as cross examination and comparison of these natural products on a same platform under exactly the same experimental conditions have not been conducted. In addition, whether administration of these natural antioxidants could increase the efflux of cadmium out of the body remains to be investigated.

## 8. Other Potential Interventional Approaches

In addition to the plant extracts, herbs and pharmacological agents as shown in Table 1, there are other approaches that have also been applied to counteract cadmium-induced kidney injury. For example, caloric restriction as an established interventional approach for aging and age-related diseases [187,188,189,190] has been demonstrated to mitigate cadmium-induced renal toxicity and kidney dysfunction [191]. Dietary restriction of calcium intake has also been shown to enhance cadmium-induced expression of metallothionein, which could minimize cadmium toxicity [192]. The protective effects of preconditioning and postconditioning observed in numerous studies [193,194,195], if any, elicited by a variety of approaches including ischemia, hypoxia, chemicals or pharmacological agents are yet to be investigated. Additionally, metal chelation using specific chelating agents may also be considered as an interventional approach [196]. Recent findings that persulfide and polysulfide can bind to cadmium thereby decreasing cadmium toxicity [197,198,199] may also provide potential approaches for counteracting cadmium-induced kidney injury.

## 9. Postulated Model of Cadmium-Induced Proximal Tubule Lesion

**Figure 6:**
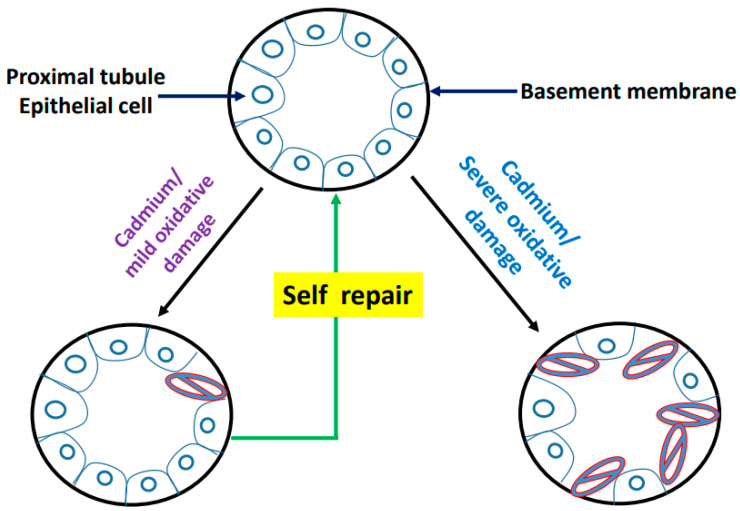
Schematic diagram depicting cadmium-induced injury to proximal tubular epithelial cells. When oxidative damage is mild, the cells can mobilize their repair defense system and self-repair, leading to maintenance of cellular function. When oxidative damage is severe, cells lose their self-repair capacity and die, leading to widespread cell death and kidney injury. (Adapted from reference [50]).

Prozialeck and Edwards proposed a model of proximal convoluted tubular cell injury in 2012 [50] that we think elaborates very well the mechanism of cadmium-induced kidney injury in terms of oxidative damage as a unifying mechanism. This model is similar to what has been proposed to explain the mechanisms of ischemic acute kidney injury [8,10,200,201]. Essentially, as diagramed in Figure 6, under healthy conditions and in the absence of cadmium deposit and accumulation, epithelial cells in the proximal tubule are closely associated with each other via specialized junctional structures. These epithelial cells align orderly and tightly on the tubular basement membrane via local adhesion molecules to collectively achieve filtration and reabsorption. In the presence of cadmium, which can accumulate in the cytosolic and mitochondrial compartments, cadmium binds thiol-containing proteins and peptides, leading to functional impairment of these cadmium-bound proteins and peptides. Consequently, such impairments cause mitochondrial electron leakage or NADPH oxidase dysfunction, resulting in enhanced production of ROS and elevated levels of oxidative stress. If the oxidative stress is mild, the tubular cells can repair themselves and resume normal function. It should be noted that this self-repair is likely achieved by de-differentiated tubular epithelial cells instead of differentiated and fixed tubular progenitor cells [202,203]. However, if the oxidative stress is severe and overwhelms cellular repair capacity, an irreversible damage process occurs and cells die by means of apoptosis, necrosis or both [204,205], leading to cell-cell and cell-basement membrane dissociations. This would lead to proteinuria, polyuria, and a progressive decline in kidney function. This functional decline, however, may be intervened and halted by the antioxidative approaches shown in Table 1 if applied appropriately. It should be pointed out that in order to distinguish cadmium-induced proteinuria from primary glomerular lesion, the magnitude of proteinuria and a cadmium concentration dependent manner will need to be characterized.

## 10. Diagnosis of Cadmium-Induced Kidney Injury

While diagnosis of cadmium-induced kidney injury is complicated by factors such as dosage of exposure, duration of exposure, early stage injury or late stage irreversible injury as well as whether there does any exist underlying disease, a series of parameters could be combined to indicate whether a kidney injury is caused by cadmium exposure. These parameters include measurements of blood and urine cadmium, urinary metallothionein, urinary β2-microglobulin and *N*-acetyl-β-glucosaminidase. In addition, kidney injury molecule-1 (Kim-1) could also be used to indicate early stage of cadmium-induced proximal tubular injury [50]. Moreover, severe cadmium poisoning could also cause pains in the spine and joints [24]. Collectively, measurements of these biomarkers or indices should provide good evidence that a cadmium-caused kidney injury has occurred. It should be noted that once these biomarkers appear and are detectable, cadmium induced kidney injury might be at an advanced stage that is irreversible. Therefore, novel biomarkers of cadmium-induced early stage kidney injury remain to be explored.

## 11. Summary

Cadmium exposure and cadmium-induced kidney disease are major public health issues. Mitochondria and NADPH oxidase can be impaired by cadmium that accumulates in the proximal tubular site of nephrons, and thus, are the major sources of ROS. Therefore, the main underlying mechanism of cadmium renal toxicity is enhanced oxidative stress and associated damage to DNA, proteins, and lipids, eventually leading to cell death, kidney injury, and decline in kidney function (Figure 5). Given that cadmium exposure is inevitable in the foreseeable future, cadmium-induced animal models of kidney disease should continue to play an important role in investigating the etiological, pathological, pharmacogenetic, pharmacological, and therapeutic aspects of cadmium-induced kidney disorders. Finally, in addition to elucidating the detailed mechanisms of cadmium-caused mitochondrial oxidative stress and redox imbalance, future studies should also explore novel biomarkers that can be used to diagnose early kidney injury by cadmium exposure. Moreover, whether administration of natural products such as those listed in Table 1 could increase the efflux of cadmium out of the body remains to be investigated.

## Figures and Tables

**Table 1:** Counteracting effects of exogenous compounds such as plant extracts, herbs, chemicals and pharmacological agents on cadmium induced renal toxicity.

Plant/Extract/Chemical	Rodent Model	Mechanism	Reference
*Allium hirtifolium boiss*	Rats	Anti-oxidative stress	[125]
Apple juice	Rats	Anti-oxidative stress	[126]
*Arctium lappa*	Rats	Anti-oxidative stress	[127]
Carnosic acid	Mice and cells	Anti-oxidative damage	[128]
Catechin	Rats	Anti-oxidative damage	[129]
Caffeic acid phenethyl ester	Rats	Anti-oxidative stress	[130,131]
*Chelidonium majus* leaves	Rats	Antidiuretic	[132]
*Chorella pyrenoidosa*	Rats	Antihyperglycemic	[133]
*Cleistocalyx nervosum var. paniala*	Rats	Increasing antioxidation power	[134]
*Coriandrum sativum* leaf	Mice	Anti-oxidative stress	[135]
Curcumin	Rats	Anti-oxidative stress	[136]
Edaravone	Mice/Cells	Inhibiting oxidative stress	[137]
Elderberry	Rats	Increasing antioxidant enzymes	[138]
Epigallocatechin-3-gallate	Rats	Increasing antioxidant defense	[139]
*Eucommia ulmoides* bark	Rats	Anti-oxidative damage	[140]
Ferulic acid	Rats	Anti-oxidative stress	[78]
*Fragaria ananassa*	Rats	Anti-oxidative stress	[141]
*Ginger*	Rats	Decrease lipid peroxidation	[142]
Glutathione	Rats	Anti-oxidative stress	[143]
Glycyrrhiza glabra	Rats	Anti-oxidative stress	[144]
Grape seed procyanidin	Mice	Antioxidants	[145]
Grape skin/purple carrot	Rats	Anti-oxidative damage	[146]
Green/black/red/white tea	Rats	Anti-oxidative damage	[147]
Green olive leaf	Renal cells (MCD4)	Anti-oxidative stress	[148]
Herbal adaptogens	Chicken	Anti-oxidative damage	[21]
*Ipomoea aquatic/Enhydra fluctuans*	Mice	Anti-oxidation/anti-apoptosis	[149]
*Irvingia gabonesis* stem bark	Rats	Increasing antioxidant defense	[150]
Licorice	Rats	Anti-oxidative damage	[151] **
Ligustrazine	Rats	Restoring renal function	[152]
Lipoic acid	Rats	Anti-apoptosis	[68,153]
Onion/garlic	Rats	Anti-oxidative stress	[154]
*Origanum majorana L*.	Rats	Anti-oxidative damage	[155]
*Persea americana* seeds	Rats	Mitigating oxidative stress	[156]
*Physalis peruviana L*	Rats	Anti-oxidation/Anti-apoptosis	[157]
*Picroliv*	Rats	Anti-oxidative stress	[158]
Plantamajoside	Rats	Decrease oxidative damage	[159]
*Pleurotus ostreatus*	Rats (female)	Mitigating oxidative damage	[160]
Potentilla anserine	Mice and cells	Anti-oxidative stress	[161]
Puerarin	Rat proximal tubule cells	Restoring mitochondrial function	[162]
Quercetin	Rats	Suppressing ER stress	[163]
Resveratrol	Chickens	Anti-oxidative stress	[164]
Roflumilast	Rats	Increasing antioxidant defense	[165]
Rosmarinic acid	Mice	Anti-oxidative damage	[166]
Royal jelly	Mice (male)	Antioxidation/Nrf2 activation	[39]
Rutin	Rats	Inhibiting oxidative stress	[167]
*Salvia officinalis*	Rats	Anti-oxidative damage	[168]
*Salvia miltiorrhiza*	Rats	Anti-oxidative injury	[169]
*Sana Makki*	Rats	Anti-oxidative stress/Nrf2	[170]
Selenium yeast	Chicken	Mitigating necroptosis	[171]
Sesamol	Rats	Inhibiting oxidative stress	[172]
SInapic acid	Rats	Inhibiting oxidative stress	[173]
*Solanum torvum Swartz*	Rats	Anti-oxidative stress	[174]
*Spinacia oleracea* polysaccharides	HEK293 cells	Anti-oxidative stress	[175]
Telmisartan	Mice	Suppressing oxidative stress	[176]
Tetrahydrobiopterin	Rats	Maintaining mitochondria integrity	[177]
*Thunbergia laurifolia* leaf	Kidney cells	Increasing antioxidant enzymes	[178]
Thymus serrulatus essential oil	Rats	Anti-oxidative stress	[179]
Thymoquinone	Rats	Increasing glutathione	[180]
*Tinospora cordifolia*	Rats	Anti-oxidative stress	[181]
Trehalose	Rats	Inhibiting oxidative stress	[182]
*Tribulus terrestris* linn	Rats	Anti-oxidation	[183]
Vitamin C	Rabbits	Anti-oxidative stress	[184]
Vitamin E	Rats	Enhancing antioxidant defense	[185]

** Please note that licorice could also pose renal toxicity under certain conditions [186].
